# Letter from B. P. Reese, M.D., of Stanton, Va.

**Published:** 1874-04

**Authors:** B. P. Reese

**Affiliations:** Stanton, Va.


Stanton, Va., March 6th, 1874.

*Messrs. Editors:*

On the night of the 12th of July, 1873, I was called to visit Mrs. C., and found her to all appearances in active labor. Upon digital examination I was a little surprised at first in not finding the os uteri, but only a smooth surface presenting, but upon further examination I discovered the womb was in an antiverted position with the os high up under the promontory of the sacrum. It felt somewhat patulous but not dilated. The pains were severe and frequent without seeming to make any impression in the progress of labor. I remained with the case several hours, and left with the promise to return early next morning. On my return I found the case in about the same condition as when I left, pains frequent and severe. I made her change position several times without accomplishing anything, also tried to push the uterus towards its normal position but could not. During the day the pains became less frequent and severe but towards night they began to get worse, and I flattered myself with the hope that all would be over before the following morning. The pains continued through the night very severe and frequent, preventing her from sleeping, although under the influence of an anodyne. I applied locally belladonna to the mouth of the womb and placed her in every conceivable position with no advantage gained. She begged from the beginning for ergot, stating that in her former confinement, five years before, it was the only thing that did her any good, but the pains were so severe I did not feel justified in using it, and substituted chloroform without satisfactory result. As there was no constitutional disturbance demanding interference, I concluded to wait the effects of nature visiting her from time to time and watching the case. She continued to beg for the ergot, and becoming impatient implored me to cut the child from her, that she had rather run the risk of her life in the operation than to suffer as she did. Her importunities became so urgent, that her husband came to me and said, something must be done to relieve his wife. I told him that I was unwilling to interfere until I saw the necessity for it, but to satisfy him I would call in advice. I took with me to see the case, Dr. Rob. S. Hamillir, who upon examination advised to wait awhile, which we did for several days, she in the mean time begging for the ergot. Finally as labor did not seem to progress at all, we concluded to commence the ergot in small doses and watch the effect. So soon as we had given sufficient to have any effect, we found the cervix was descending and the os dilating.

About this time Dr. H. was called away and I remained with the case, which gradually went on to a natural and favorable termination, giving birth to a small female living child, on the 12th day of August, which is now living and is a fine child. I omitted to state that the mother is paralyzed on the left side, and has been since early childhood.

Here is a case to all appearances in which the ergot was contra-indicated, and yet in the administration of it, we had the happiest effects—I have several theories in my own mind how the ergot acted beneficially in this case, but as my object was only to report the facts of the case, but time and space forbid my saying more at present. I conclude by requesting that if any of your large number of contributors should have any suggestions, I would be pleased to hear from them. B. P. Reese, M.D.

